# Risks and benefits of transfusion for children with severe anemia in Africa

**DOI:** 10.1186/1741-7015-12-68

**Published:** 2014-04-25

**Authors:** Thomas Brick, Mark J Peters

**Affiliations:** 1 Paediatric Intensive Care Unit, Great Ormond Street Hospital NHS Foundation Trust, Great Ormond Street, London WC1N 3JH, UK; 2 Respiratory Critical Care and Anaesthesia Unit, Institute of Child Health, University College London, 30 Guilford Street, London WC1N 1EH, UK

**Keywords:** Africa, Physiology, Anemia, Children, Commentary, Infectious disease, Malaria, Transfusion

## Abstract

Severe anemia contributes significantly to child mortality in sub-Saharan Africa. Blood transfusion is used in emergencies but carries risks. In *BMC Medicine,* Olupot-Olupot and colleagues report the findings of a phase II trial in children with severe anemia in Eastern Uganda. They provide important early safety and efficacy data supporting large volume whole blood transfusion (30 ml/kg) compared with the World Health Organization recommendation of 20 ml/kg. Large volume transfusions result in more rapid and frequent correction of severe anemia; they can be expected to reduce the risk of transfusions, and help manage the scarce resource of donor blood. However, severe anemia arises from varying combinations of acute, sub-acute and chronic etiologies.

The Fluid Expansion As Supportive Therapy study reminds us that the risks and benefits of even simple interventions are complex, and that rapid normalization of physiology may not always be the best strategy. There is no substitute for high quality evidence and to this end we strongly support Olupot-Oluput and colleagues’ call for a definitive trial of large volume transfusions in severe anemia.

Please see related research article http://www.biomedcentral.com/1741-7015/12/67/abstract.

## Background

An estimated 6.6 million children died worldwide in 2012. The highest rates of child mortality are found in sub-Saharan Africa where 98 per 1,000 children die before the age of five. This represents nearly half of the global deaths in children under five. Child mortality has fallen by 45% since 1990, although at projected rates of decrease, the Millennium Development Goal Four - a two-thirds reduction in the under-five mortality rate by 2015 - will not be met [1].

Anemia is responsible for 11% to 14% of childhood deaths in highly malarious regions of sub-Saharan Africa [2]. Between 12% and 29% of children admitted to hospital in these areas are severely anemic [3] (often defined as a hemoglobin level <5 g/dL). The outcomes for severe anemia are poor: in-hospital mortality is between 6% and 16% [4-7] with a longer-term mortality of 13% to 30% [5,8]. The etiology of severe anemia varies by region; malaria predominates in endemic areas. Other contributing factors include malnutrition, vitamin deficiency and infections with bacteria, human immunodeficiency virus (HIV), hookworm and schistosomiasis. The interaction between these risk factors is complex [3,9].

### Blood transfusion in sub-Saharan Africa

Blood transfusion increases short-term survival from severe anemia with respiratory distress [4,8] but two thirds of deaths occur before a blood transfusion is administered [10]. There is currently no evidence of a long-term survival benefit of transfusion. This may reflect difficulty in addressing the underlying parasitemia, bacterial infection or malnutrition, or might mean that current transfusion practice is sub-optimal [4,8].

### Large volume blood transfusions

In a recent phase II trial published in BMC Medicine, Olupot-Olupot and colleagues provide a welcome step towards obtaining a definitive answer on the choice of blood transfusion volumes in severe anemia in sub-Saharan Africa [11].

Olupot-Olupot *et al.* provide pilot safety and efficacy data of a large volume transfusion (30 ml/kg) of whole blood in children with severe anemia in comparison to the current World Health Organization (WHO) guideline of 20 ml/kg [12]. The children in this study were sick: half were in respiratory distress, one third were prostrate and one third had a blood lactate level >5 mmol/l. All received a bundle of standard care. The large volume transfusion arm demonstrated more frequent correction and higher resultant hemoglobin than the 20 ml/kg arm; an effect that was sustained at 48 hours, although not at 28 days. At 28 days, the overall mortality was 4%. This compares favorably with 8% to 30% case fatality in other studies [4,8], and supports rapid access to transfusion and a standardized bundle of care in children with severe anemia in sub-Saharan Africa.

### The risk-benefit relationship for blood transfusion

The justification for this study is two-fold: full resuscitation is likely to be more effective than partial resuscitation, but - perhaps more importantly - multiple transfusions in this environment are problematic. Blood transfusion in sub-Saharan Africa carries risks and is a scarce resource. Under-resourced laboratory facilities and poorly trained clinical and laboratory staff contribute to this risk. The last decade has seen the introduction of national transfusion services with the aim of ensuring a supply of ‘safe blood’. The impact of these changes is not fully understood and the provision of safe blood remains a considerable challenge [13]. Viral transmission rates from blood transfusion in sub-Saharan Africa are significant. The risks of HIV, hepatitis B virus and hepatitis C virus infection are estimated at 1, 4.3 and 2.5 cases per 1,000 units of blood transfused, respectively [14]. Blood is frequently unavailable, and therefore is often donated as an emergency for urgent transfusion [15]. The WHO guidance on blood transfusion promotes the rational use of this scarce, and potentially dangerous, resource by advocating a restrictive approach to blood transfusion in severe anemia [12].

Increasing the volume of blood transfusion from a single donor is therefore a potentially important strategy. It offers the possibility of increasing the efficacy of transfusion without the additional risk of multiple donor exposures and opportunities for errors. Olupot-Olupot *et al.*[11] observed an increased efficacy of transfusion (90% of children have anemia corrected by 24 hours with 30 ml/kg versus 74% with 20 ml/kg; risk ratio (RR) 1.54, 95% confidence interval (CI) 1.09, 2.18) alongside a trend towards a reduction in meeting criteria for subsequent transfusion (RR 0.35, 95% CI 0.12, 1.04). A trend towards reduced early (<48 hour) and 28-day mortality is encouraging. This combination of results makes a powerful case for a definitive trial.

Should practice change on these data alone? After all, there is a sound physiological basis for improving oxygen delivery by raising hemoglobin whilst minimizing the risks of treatment. We suggest that the answer is ‘not yet’. This caution arises, perhaps ironically, from the same group’s previous work. The unexpected results of the remarkable Fluid Expansion as Supportive Therapy (FEAST) study [16] - in which volume expansion was inferior to ‘no bolus’ for children with severe febrile illness - may well be relevant. At the very least these results caution against deriving treatment strategies by simply correcting physiological variables. A process of protective physiological adaption may have taken place in response to disease over acute, sub-acute or chronic time-scales and rapid correction of physiological variables must be undertaken with caution. Supplementary analysis of the FEAST data has revealed the finding that excess mortality due to rapid boluses of intravenous fluids was primarily due to cardiovascular collapse, not cerebral edema or congestive heart failure as had been anticipated [17]. In environments without ready access to oxygen, continuous positive pressure ventilation or inotropic support, the principle of cautious physiological correction applies perhaps more strongly than elsewhere.

Olupot-Olupot *et al.* understand the need for close monitoring for fluid overload; indeed, clinical signs of fluid overload were specified as a serious adverse event (SAE). It is reassuring that SAEs were not greater in the 30 ml/kg group and that none of the seven fatal events were recorded as being due to heart failure. However, the mechanism by which blood transfusion is beneficial whilst volume expansion is detrimental in sick children with anemia still needs to be elucidated.

## Conclusions

The threshold at which to transfuse blood in children with severe anemia in sub-Saharan Africa is determined by a number of factors that affect the risk-benefit relationship. Some of these can potentially be quantified: the risk of viral transmission through blood, the more general risks of blood transfusion, the risk of rapid volume expansion in the critically ill child, the risk of small volume transfusion not achieving benefit, the resource cost of a transfusion service, and the opportunity cost of foregoing alternative healthcare interventions. We can, cautiously, hypothesize that the benefit of transfusion increases both as the child’s hemoglobin falls and when severe anemia is accompanied by severe physiological derangement. The risk-benefit relationship of transfusion is likely to be context specific, and will be influenced by region, etiology of severe anemia and accompanying disease. The complexity of these factors is such that definite empirical data from a large-scale phase III trial is the way forward. Oluput-Oluput and colleagues have moved this closer with their important study.

## Abbreviations

CI: confidence interval; HIV: human immunodeficiency virus; RR: risk ratio; SAE: serious adverse event; WHO: World health organization.

## Competing interests

The authors declare that they have no competing interests.
